# Hospital Festivals and Meetings

**Published:** 1894-02-17

**Authors:** 


### HOSPITAL MEETING.

### ST. GEORGE'S HOSPITAL.

The quarterly general court of the governors of St. George's Hospital was held on Friday, the 9th, with Mr. H. M. Macpherson in the chair. Among those present were Lord Saltoun, Admiral Sir G. O. Willes, K.C.B., the Hon. L. Agar-Ellis, the Rev. Canon Heathcote, and Mr. J. R. Mosse (treasurer). The report of the weekly meetings since the last quarterly court having been read and approved, four new governors—the Marquis of Zetland, Colonel H. B. Hamilton, the Hon. T. A. Brassey, and John Aird, jun., Esq.—were elected. The certificates to the outgoing house physician and house surgeon having been read for the approval of the court, Admiral Sir G. O. Willes expressed his opinion that a certificate granted after only six months' work was not of much value. Mr. J. R. Mosse replied that as the posts of house physician and house surgeon were only open to students of the hospital, the certificates were, in reality, only granted after several years work and experience there. The number of in-patients during the last quarter was stated to be 1,154; of out-patients, 8,181. The subscriptions for the same period had shown a net decrease of £144 10s, the new subscriptions amounting only to £57, while the loss from death and withdrawal of subscribers was £201 10s.

